# Portable PET probes are a novel tool for intraoperative localization of tumor deposits

**DOI:** 10.1186/1750-1164-3-2

**Published:** 2009-02-21

**Authors:** Vivian E Strong, Charles J Galanis, Christopher C Riedl, Valerie A Longo, Farhad Daghighian, John L Humm, Steven M Larson, Yuman Fong

**Affiliations:** 1 Department of Surgery, Memorial Sloan-Kettering Cancer Center, New York, USA; 2 Department of Radiology, Memorial Sloan-Kettering Cancer Center, New York, USA; 3 Department of Research Animal Resource Center, Memorial Sloan-Kettering Cancer Center, New York, USA; 4 IntraMedical Imaging, Los Angeles, USA; 5 Department of Medical Physics, Memorial Sloan-Kettering Cancer Center, New York, USA; 6 Department of Nuclear Medicine, Memorial Sloan-Kettering Cancer Center, New York, USA

## Abstract

**Background:**

Positron emission tomography (PET) identifies cancer deposits by detecting sites of gamma emissions that are released from radioactively labeled molecules targeting tumor to formulate a PET image. Correlating preoperative PET scans with intraoperative findings remains a challenge. We investigated whether high-energy gamma emissions detected by a novel hand-held PET probe would detect tumors and offer a real-time method to localize tumor intraoperatively. Furthermore, we investigated the novel beta probe, which detects emissions at a shorter range than gamma emissions, making them undetectable by PET scanners, but potentially valuable for close range intraoperative detection of tumor deposits.

**Methods:**

Six-to-eight-week-old athymic mice were injected with one of four possible tumor cell lines: gastric, pancreas, squamous cell and breast cancer. After tumors reached at least 1 cm in size, they were euthanized and imaged with a micro-PET imager. Hand-held gamma and beta probes were then used in vivo and ex vivo to measure high-energy gamma and beta emissions.

**Results:**

The portable PET probes detected high-energy gamma and beta emissions from all tumors evaluated. These emissions were reproducible and we established that beta emissions correlate with high-energy gamma emissions and conventional PET scans. There was a strong positive correlation (R = 0.8) between gamma and beta counts. Beta emission showed a stronger correlation than gamma emission with overall tissue radioactivity.

**Conclusion:**

This study is the first to demonstrate that gamma emission detected by conventional PET imaging correlates with beta emissions. This study shows that compared to detection of gamma emissions, beta counts may offer superior real-time localization of tumor deposits. Intraoperative portable PET probe may become a useful way to exploit tumor biology and PET technology to guide real-time tissue characterization during surgery.

## Background

Positron emission tomography (PET) scans using fluorodeoxyglucose (FDG), an analog of glucose labeled with the positron-emitting fluorine-^18^, is based on the recognition that malignancies accumulate FDG at greater rates than normal tissue [1]. PET scans have helped increase accuracy of identifying occult sources of cancer and to improve the staging of patients with potentially curable cancer by finding distant sites of tumor spread. However, difficulty remains in pinpointing specific sites of tumor and identifying small cancer deposits as the resolution of PET scans is at best about 1 cm [2]. Unsuspected intra-abdominal disease is still frequently first detected at the time of surgery [3]. Diagnostic laparoscopy is indicated for staging and to determine resectability prior to extensive tumor resections, however, laparoscopy is limited by grossly visible tumor and thus the surgeon may miss occult sites of tumor by leaving unseen tumor behind [4,5]. Furthermore, a tool that could identify PET avid sites in the operating room during surgery on the peritoneal surface, in lymph nodes, or after resection of tumor to confirm negative margins, would provide a valuable tool for surgeons.

Currently, no hand-held tool, other than the one described here, is able to detect both high-energy gamma emissions like a PET scan does and beta emissions. This beta probe detects radioactive emissions that a PET scanner does not detect, partly because beta emissions only travel millimeters from the source of radioactivity and can not be detected by a PET scanner. Although this property makes them undetectable by external imaging (i.e. PET), beta emissions are an ideal target for intraoperative detection of tumor at close range and are a novel and potentially useful tool for an oncologist during surgery.

A beta detector probe has been recently developed that allows for detection of beta rays emitted by the isotopes used for conventional PET scanning [6]. This probe is made from a thin crystal sufficient to stop electron radiation but too thin to be sensitive to gamma rays. This provides a promising means to detect intraoperative local positron emission in the form of beta particles. Detection of these particles would exhibit much higher local specificity over gamma radiation and additionally would allow for minimally invasive intraoperative detection that could provide superior detection of small tumor deposits during surgery with precision. If we can prove that beta detection is clinically equivalent to high energy gamma detection for identification of PET tracers and tumor, we will have validated a new tool that can improve radionuclide-guided surgery.

Our hypothesis is that if shorter range beta emissions are detectable with this novel device, then gamma emissions detected by a hand-held gamma probe should correlate directly with beta emissions from a hand-held beta probe. In this study, we aim to investigate the characteristics of beta emission detected with hand-held probes in comparison to detected gamma emissions and then characterize these emissions in a biologic model, with various tumors implanted in mice. Murine tumor models included breast, gastric, squamous cell, and pancreatic cancer. Mice were injected with ^18^F-FDG and micro-PET imaging and portable PET probe measurements of gamma and beta emissions from tumor and normal tissue were correlated.

## Materials

### Animal tumor model

The murine studies described all comply with the regulatory requirements of the Institutional Animal Care and Use Committee (IACUC), the Research Animal Resource Center (RARC) of MSKCC, and the National Institutes of Health (NIH) "Guide for the Care and Use of Laboratory Animals".

Four human cancer cell lines were studied: the breast cancer line MCF-7, the gastric cancer line OCUM, the pancreatic cancer line Panc-1, and the head and neck squamous cell carcinoma line SCC15. The OCUM gastric cancer cells were a gift of Masakazu Yashiro (Osaka City University Medical School Osaka, Japan) and were maintained in DMEM with high glucose, 2 mM L-glutamine, and 0.5 mM sodium pyruvate. All other lines were obtained from the American Type Culture Collection (Rockville, MD) and grown in the recommended media. Cells were maintained in a 5% CO_2 _humidified incubator at 37°C and subcultured twice weekly.

Six to 8-week old female athymic mice (20–25 g) were housed four per cage and allowed food and water *ad libitum*. All animal procedures were performed under anesthesia by inhalation of 2% isoflurane. Animals were killed by CO_2 _inhalation. Tumors were established by injecting ~1 × 10^6 ^cells in 50 μl PBS into the subcutaneous flanks. Each animal was injected with one tumor for a total of 15 tumors injected. 5 animals were euthanized by the animal facility to assure animal comfort. Animals were studied once the tumor diameter reached 1 cm at a time of 4–6 weeks. Tumor growth was checked every second to third day.

### PET probes

The high energy gamma and beta probes (IntraMedical Imaging LLC, Los Angeles, CA) are designed to detect 511-keV photons from positron-emitting sources (gamma probe) and positrons (or beta rays) directly (beta probe). The high-energy gamma and beta probes were calibrated to accurately localize the point source of ^18^F-FDG and the count rate was determined to optimize the detection of the 511 keV emissions. Radioactive emissions were measured in counts per second and recorded in triplicate.

### Radioisotope production and injection into mice

^18^F-FDG was obtained from the institutional radiopharmacy laboratory (Nuclear Medicine Department, MSKCC, New York, NY). Tail vein injections of 5 uCi of ^18^F-FDG suspended in 0.5 cc sterile PBS were conducted under anesthesia.

### MicroPET and CT imaging

Mice were imaged 1 hour after injection with ^18^F-FDG. Animals were imaged in prone position using either the R4 or Focus 120 microPET™ dedicated small-animal PET scanners (Concorde Microsystems, Knoxville, TN). With transaxial fields of view of 10 cm and s axial The transaxial field of view covered the lower half of the thorax (including the heart) and the upper half of the abdomen. Scans were collected with an energy window of 350–750 keV and a coincidence timing window of 6 nsec. Data was sorted into 2D histograms by Fourier re-binning and transverse images were reconstructed in a 128 × 128 × 63 (R4) or 128 × 128 × 96 (Focus 120) matrices by filtered back-projection. Images were corrected for non-uniformity of scanner response, and radionuclide decay to the time of injection. For anatomical orientation on the PET images, CT scans were performed on a dedicated small-animal CT scanner (X-SPECT, Gamma Medica, Northridge, CA).

### PET probe measurements from mice and tissue preparation

Following imaging, all 10 animals (with pancreatic, gastric, breast and squamous cell carcinoma tumors) were sacrificed and tumors were harvested *en bloc *with their surrounding tissue. Size measurements were done both in vivo and ex vivo after tissue had been excised. Beta and gamma measurements were done for each tumor and for background levels in triplicate. The amount of radioactivity from the 18F-FDG source was measured via a scintillation counter to control for the amount of radiation used per mouse. Tissue samples including heart, kidney, liver, bowel, and muscle were then isolated and radioactivity measured with the hand-held PET probes for both gamma and beta emission. All counts were repeated in triplicate and counts represent the average value obtained. All counts were obtained with a dwell time of 0.5 seconds.

Tissue samples were then frozen in dry-ice and isopentane. Frozen tissue sections of 10 μm thickness were cut from various levels of the specimens and mounted on poly-L-lysine slides (Fischer Scientific, Pittsburgh, PA). Slides were placed onto an imaging phosphor plate for autoradiography and kept stored at -20°C. Three days later, the images were read out at 100 μm resolution using a phosphor-plate reader (Model G-350; Bio-Rad Laboratories, Hercules, CA). Subsequently, Hoechst distribution was assessed on the identical tissue sections. For this, up to 332 images of tumors up to 2 cm in diameter were acquired using a 5× magnification on a Zeiss Axiovert 200 M inverted stand microscope (Carl Zeiss, Inc., Oberkochen, Germany). The images were montaged using Metamorph 6.2r3 software (Universal Imaging Corporation, Downingtown, PA). Finally, the slides were stained with H&E, imaged under bright field, and again montaged.

### Image analysis

PET image analysis was done with ASIPro™ software (Concorde Microsystems Inc., Knoxville, TN). To verify ROI measurements, selected tissues were harvested, weighed, and counted in a scintillation well counter calibrated for ^18^F-FDG.

## Results

**Figure 1:**
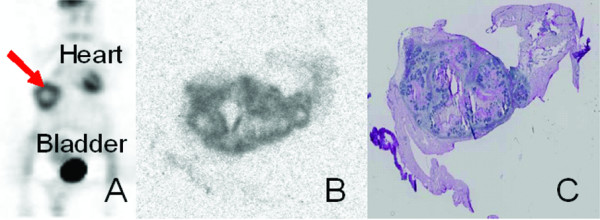
**Conventional PET imaging detects gamma waves emitted from tumors in a murine model**. Flank tumors of multiple tumor types (breast, pancreatic, gastric, and squamous cell carcinoma) were grown in nude mice. Mice were injected with radiolabeled ^18^F and imaged 1-hour later by a microPET scanner. Animals were then sacrificed and tumors were sectioned for analysis by autoradiography and hematoxylin and eosin (H&E) staining. Shown is a representative animal PET scan with tumor highlighted by a red arrow (A), autoradiograph of tumor section (B), and H&E of tumor section (C).

The results in Figure 1 are representative PET images along with the autoradiographic image and corresponding H&E staining of excised tumor specimen obtained from flank tumors of multiple tumor types, including pancreatic, gastric, squamous cell and breast cancer. Panel A shows a micro PET image from a mouse with gastric carcinoma (demonstrated by arrow). Other areas of visualized uptake in the micro PET images include the heart and bladder (labeled).

Panel B and C show the autoradiographic image and H&E section for the same tumor.

**Figure 2:**
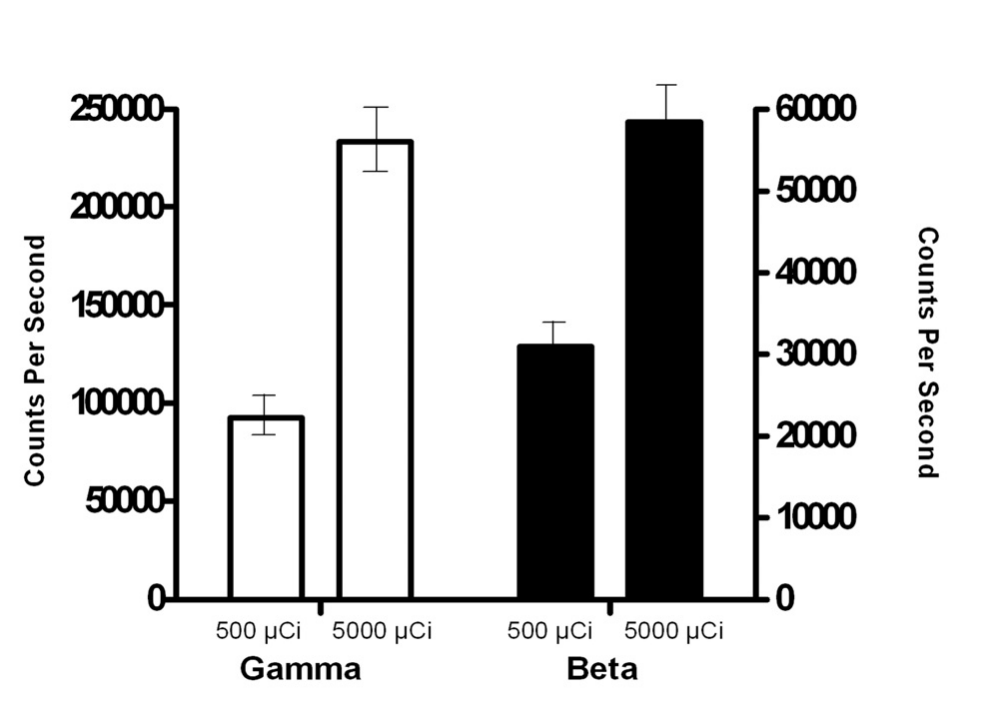
**Portable PET probes detect gamma emission and beta emission from both low and high tested doses of radiation *in vitro***. Multiple samples of radiolabeled ^18^F were obtained at two doses (500 μCi and 5000 μCi), Measurements were taken using the portable probes for both gamma and beta emission at the source and at distances up to 5 cm from the source.

In order to establish detection of gamma and beta emissions from low and high doses of radioactivity, two doses at 500 μCi and 5000 μCi were evaluated (Figure 2). Multiple samples of ^18^F-FDG were tested at the two doses and measurements were taken using the portable PET probes for both gamma and beta emissions at the source and at distances up to 5 cm from the source. There was a significant difference in counts per second obtained from the gamma and beta probes at both the low and high doses.

**Figure 3:**
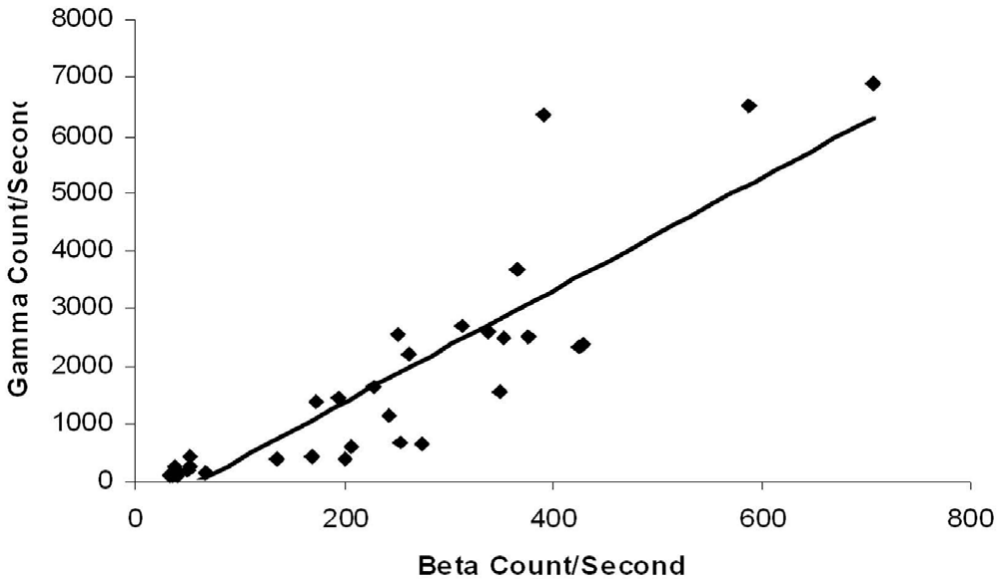
**Beta emission directly correlates with gamma emission in all tested tumor types *in vivo *(R = 0.8)**. Four tumor types were tested for gamma and beta emission following animal injection with radiolabeled ^18^F (n = 10 animals). Pancreatic, gastric, squamous cell and breast cancer cell lines injected subcutaneously into the murine model. Emissions were measured with portable probes on the tumor *in vivo *as well as on excised tumor *ex vivo *once tumors reached 1 cm in size. Beta and gamma emission directly correlated as determined by the Pearson correlation calculation of the R value.

To demonstrate the relationship of gamma to beta counts in the in vivo model, measurements were graphed from all counts obtained from the four tumor types studied in the 10 mice. Figure 3 shows a strong positive linear correlation (R = 0.8) for the *in vivo *model comparing gamma and beta emissions.

**Figure 4:**
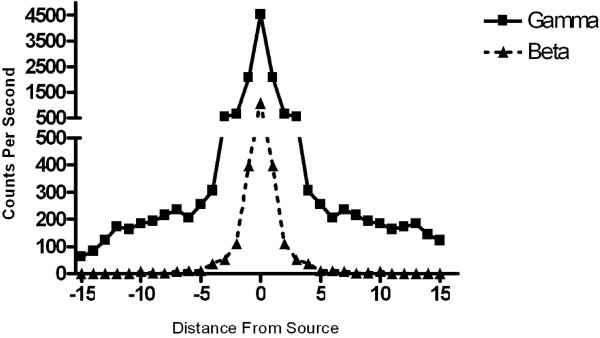
**Beta detection better pinpoints the source of radiation than gamma detection**. Beta and gamma measurements were taken at incremental distances from the tumors 1-hour following intravenous injection of radiolabeled ^18^F. Beta detection was nearly zero at distances > 5 cm from the source, while gamma emission was still detectable at 15 cm. Data from a representative animal is shown to demonstrate that at the spot over tumor, the beta counts are high, but after the probe is moved ~2 cm away from the tumor, counts are nearing zero. In contrast, the gamma probe detects emissions up to 10 cm away from the site of tumor.

To characterize the range of localization of the high-energy gamma probes in comparison to the beta probe, measurements were taken at incremental distances from mouse flank tumors one hour following injection of radiolabeled ^18^F. Beta detection was nearly zero at distances greater than 5 cm from the tumor, whereas high-energy gamma emissions were still detectable at 15 cm from tumor (Figure 4).

**Figure 5:**
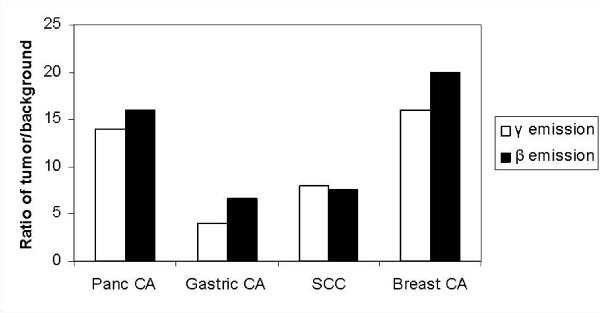
**Ratio of gamma and beta counts to background counts *in vivo *in mice with pancreatic, gastric, breast, and squamous cell tumors**.

Figure 5 demonstrates the ratio of tumor over background counts in the mouse for gamma and beta emissions. Results for each tumor type were averaged for all mice studied in each tumor group. For the mice with pancreas tumors, gamma emissions were detectable at 14 times background counts and beta emissions at 16 times background counts. Gastric tumors in mice demonstrated ratios with 4-fold over background for the gamma and 6.7-fold over background for the beta emissions. Squamous cell tumors demonstrated an average gamma to background ratio of 8, and beta emissions of 7.6 over background. Gamma counts from the breast tumors were 16 times higher than background compared to 20 times above background for the beta.

## Discussion

Hand-held PET probes to detect positrons were first described and developed by Daghighian et al [6] in 1994 as a novel method to direct intraoperative tumor localization. Unlike low-energy gamma probes that allow for detection of markers such as Tc99 (used for sentinel node mapping but not for identification of tumor cells), these positron detecting devices allow for direct detection of radiolabeled tumor cells by detecting the same high-energy gamma rays (511 KeV) emitted from ^18^F-FDG that are utilized to produce a PET scan. In addition, the beta probe, allows for the direct, intraoperative detection of positrons (beta rays) at a close range (millimeters) to the tumor of interest. This technology provides a unique combination of detection devices that in a small hand-held form can be used in the operating room to detect smaller foci of tumor than possible by conventional PET scanning. In addition, the ability to use this technology as a real-time device before, during and after resection of tumor to identify additional tumor deposits, lymph nodes and clear resection margins, would provide a valuable and feasible tool to improve cancer resections.

The gamma probe, although useful for open resections, can not be made smaller than a 12 mm diameter, due to the heavy collimation needed to shield side-scatter from the high-energy particles. For this reason, the beta probe is of particular interest because of its potential to be produced in a small form that can be used through a 5 mm laparoscopic port site.

Several papers have discussed the use of the high-energy gamma probes in the operative setting, discussing their ability to help the surgeon identify tumors during procedures and results are promising [7-9]. The purpose of this study is to establish that the positron-detecting beta probe is able to detect tumor sites with equivalent reliability as the more bulky gamma probe. The results from our study demonstrate that the beta probe appears to be more specific than the gamma probe as it identifies tumor at closer ranges (within cms) and correlates reliably with readings obtained from the more conventional gamma emissions.

We found that the gamma probe is sensitive however, detects counts up to ~10–15 cm from an *in vitro *test source, while the beta probe is similarly very sensitive and additionally has a shorter range of detection from the test source. This demonstrates that the gamma probe may not be as specific in identifying smaller tumor deposits as the smaller, more precise properties demonstrated by the beta probes, due to gamma ray scatter that is detectable centimeters from the direct source. This property makes the beta detector valuable for evaluation of small tumor deposits such as lymph nodes of interest (such as in gastric cancer operations) or for peritoneal disease spread (as in pancreatic cancer). Although this study has helped to establish a range of usability for this technology the limit of detectability for the probes needs to be studied in further experiments.

To evaluate the relationship of beta and high-energy gamma counts to one another, direct counts were obtained in triplicate from various sources of ^18^F-FDG. We were able to demonstrate the relative comparison between high-energy gamma and beta emissions. We found gamma counts were roughly three to five times higher than beta counts with different *in vitro *test dose levels, establishing that beta counts have a consistent relationship to gamma counts and can be reliably used to detect radioactive tracer. This demonstrates that the probes are able to measure tumor counts from the 500 uCi to the 5000 uCi range and still maintain differentiation in measurements.

The utility of the beta probe in the biologic murine model was evaluated by testing mice implanted with four different human cancer lines; pancreatic, gastric, breast and squamous cell. We aimed to evaluate whether background interference inherent in a biologic model would alter the reliability and specificity of the gamma and beta probes. We demonstrated that gamma and beta emissions strongly correlate with a correlation coefficient of 0.8, in a manner very similar to the *in vitro *experiments. Evaluation of four tumor types in terms of the ratio of counts from the tumor itself compared to background counts from the mouse permitted calculation of ratios that are reproducible and demonstrate feasibility of using these probes intraoperatively. On average, high-energy gamma counts were between 4 and 16-fold higher than background counts over four different tumors tested and allowed for good identification of tumor sites. Beta counts were consistently 7 to 20-fold over background counts, making identification of tumor sites feasible. Interestingly, the breast cancer tumors had the highest ratio of beta counts. These animals were placed in the PET scanner an average of 80–90 minutes after injection of ^18^F-FDG compared to 60 minutes for the other tumors evaluated. It is possible that this increased interval to scanning and measurement with high-energy gamma and beta emissions, altered background levels of ^18^F-FDG compared to tumor uptake, causing an altered differential between tumor and background. This question of whether timing of gamma and beta measurement from the time of ^18^F-FDG injection alters tumor to background ratio, will be answered by future studies. The characterization of ratios of detection for the two probes in various types of tumor is very helpful as it validates the ability to detect these tumors in comparison to background uptake of radioactivity.

## Conclusion

In summary, this study demonstrates that the beta probe allows for detection of tumor and correlates with high-energy gamma probes in a reliable and reproducible manner. In addition, we have demonstrated that the beta probe may provide increased sensitivity over the gamma probe, as it may be able to identify smaller sites of tumor. The utility of this in the operating room would be paramount. Not only could real-time intraoperative detection and confirmation of tumor be possible, but evaluation of additional, unseen deposits of tumor will be possible. In this study, we were able to easily identify tumors that were implanted in this murine model, but we have yet to establish the lowest detectable limit *in vivo*. Further studies of this promising technology are warranted and will establish the role of hand-held PET probes in the resection of cancer.

## Competing interests

The authors declare that they have no competing interests.

## Authors' contributions

VS, CG, CR, VL carried out the murine studies, radiographic imaging and analysis of data. VS, CG, FD, JH, SL and YF drafted and critically revised the manuscript. VS, CG and YF participated in the design of the study.
